# Pandemic related changes in social interaction are associated with changes in automatic approach-avoidance behaviour

**DOI:** 10.1038/s41598-023-31447-5

**Published:** 2023-03-21

**Authors:** Amanda Henwood, Mike Rinck, Dario Krpan

**Affiliations:** 1 Department of Psychological and Behavioural Science, London School of Economics and Political Science (LSE), London, UK; 2 Behavioural Science Institute, Radboud University, Nijmegen, The Netherlands

**Keywords:** Psychology, Human behaviour

## Abstract

People’s natural tendencies to either approach or avoid different stimuli in their environment are considered fundamental motivators of human behaviour. There is a wealth of research exploring how changes in approach and avoidance motivational orientations impact behaviour with consequences for wellbeing. However, research has seldom explored this relationship in reverse. The COVID-19 pandemic offered a unique opportunity to explore whether widespread changes in social behaviour are associated with changes in automatic approach-avoidance tendencies over time. We gathered online survey data on people’s adherence to 7 of the prescribed social restrictions set out by the UK government and people’s automatic approach-avoidance tendencies in response to different stimuli (sad/happy faces and social scenes) at three time points during the COVID-19 pandemic. Reduced-overall-interaction (digital and in person) was found to be significantly associated with faster avoidance relative to approach of sad faces. The results suggest that automatic approach-avoidance tendencies may function to protect people against the typically negative experience of reduced social interaction, with important implications for understanding public resilience during times of crisis, and beyond.

## Introduction

According to approach-avoidance theories, all behaviour can be conceptualised as a response to appetitive (rewarding) or aversive (punishing) stimuli^1^. It is generally considered adaptive for humans to approach appetitive stimuli and avoid aversive stimuli. Changes in these approach-avoidance tendencies have been shown to characterise a host of behavioural and psychological disorders^2^. Identifying which behaviours and contexts facilitate adaptive and maladaptive approach-avoidance tendencies is therefore critical for understanding and promoting societal wellbeing.

Automatic approach-avoidance tendencies are typically captured using a joystick task (usually referred to as Approach-Avoidance Task, AAT) or a manikin task (called the Stimulus–Response Compatibility Task, SCRT)^2^. In the former, individuals must either pull the joystick to enlarge images (approach) or push the joystick to shrink the images (avoidance) on the screen in front of them. In the SRCT, participants press a computer key to make a little manikin on the screen move towards (approach) or away from (avoid) the picture. Approach-avoidance tendency scores are computed by taking the difference between the time it takes people to avoid minus approach certain stimuli presented to them. The higher the tendency score, the faster people are to approach instead of avoid a given group of stimuli. For example, the so-called “sad tendency” is people’s tendency to approach sad stimuli faster than to avoid them.

Approach-avoidance tendencies are sensitive to various situational (e.g. threat), personal (e.g. anxiety) and behavioural (e.g. forced approach or avoidance of certain things in the environment) factors. For example, socially anxious people tend to evaluate neutral stimuli as more threatening relative to healthy individuals^3^. As a result, they tend to be more avoidant of social stimuli, such as faces, than healthy individuals. Controlled approach-avoidance behaviour and subjective evaluations may differ from automatic (fast, efficient, goal-independent, and unconscious^4^) approach-avoidance behaviour. Socially anxious individuals, for instance, evaluate smiling faces just as positively as non-anxious controls. However, they show automatic avoidance of these smiling faces, being faster to avoid them than to approach them, whereas non-anxious individuals are faster to approach than to avoid them^5^. Similarly, abstinent alcohol-dependent patients avoid alcoholic beverages, and they report disliking alcohol, but nevertheless are faster to pull alcohol-related pictures closer than to push them away, unlike healthy participants^6^. This illustrates the need to study both controlled and automatic approach-avoidance behaviours, though separately from one another.

Using the joystick task or the manikin task, dysfunctional automatic approach-avoidance tendencies have also been found in cannabis and nicotine addiction, eating disorders, several anxiety disorders, and in depression^2^. The importance of these dysfunctional tendencies in psychopathology is illustrated by the fact that they can be modified by extensive joystick task trainings (a specific type of Cognitive Bias Modification, CBM), which then can lead to reduced psychopathology. In these trainings, participants usually use a joystick to complete many hundred trials of pulling closer pictures of stimuli they automatically avoid or fail to approach (as in anxiety disorders and depression, respectively). The opposite is done in addictions, where automatic drug-avoidance is trained by having patients complete many hundred trials of pushing away pictures of the relevant drug. For instance, a 6-session training during which pictures of alcoholic beverages are always pushed away with a joystick, and pictures of non-alcoholic beverages are always pulled closer, reduces relapse rates in currently abstinent alcohol-dependent individuals by about 10%^2^. Similarly, training socially avoidant individuals to approach pictures of smiling faces (which they would typically avoid), has been shown to promote more adaptive behavioural responses^7,8^. Identification and alteration of these endogenous factors has led to significant progress in understanding and promoting individual wellbeing^9^.

However, all of this research addressed existing dysfunctional approach-avoidance tendencies without being able to identify their origin (when comparing patients to healthy controls), or the tendencies were modified directly via training (when CBM training protocols were tested), or tendencies were assessed under carefully controlled laboratory conditions (when responses to conditioned stimuli were measured). In contrast, nothing is known about how significant real-world influences might be associated with the approach-avoidance tendencies shown by large, unselected samples from the general population.

The COVID-19 pandemic provided a unique opportunity to test exactly this. Since its outbreak in 2019 COVID-19 has caused more than 6 million deaths worldwide^10^. To limit the spread of the virus many governments encouraged, or in some cases enforced, considerable restrictions on social behaviours. Social distancing, self-isolation, mask-wearing in public, and reduced social interaction and gatherings were amongst the most popular of these measures. These policies facilitated a marked shift in social behaviour across the globe^11^. As such, the crisis offered the opportunity to explore the impact of widespread behaviour change on approach-avoidance tendencies. Utilising this, the present longitudinal study explored whether changes in social behaviour can be associated with changes in automatic approach-avoidance tendencies in a natural setting.

COVID-19 policy responses were determined in large part by a focus on avoiding mortality risks and deterioration of physical health^12–14^. However, to truly understand the full impact of any behavioural measures put in place to alleviate physical health risks, it is necessary to obtain a clear vision of the impact of such measures on psychological health, too. A growing number of studies have highlighted strong associations of psychological behaviours such as social distancing with mental health and wellbeing. A US study conducted in March 2020, as the pandemic grew worse and stay-at-home orders were issued, found social distancing behaviours to be associated with increases in anxiety and depression^15^. In the UK, a large (70,000) longitudinal study assessing the trajectories of anxiety and depression over the 20 weeks after lockdown was announced, found that anxiety and depression were particularly high at the start of these measures being introduced^16^. However, these high levels of anxiety and depression declined rapidly thereafter, suggesting successful adaptation to the measures over time by many. Whilst circumstantial risk factors such as being female, young, and having lower educational attainment have been explored, the processes underpinning such impacts and adaptation remain underexamined.

Approach-avoidance tendencies are an important mechanism through which reductions in social behaviours might impact wellbeing. For example, people may respond to reduced social interaction by increasing their approach relative to avoidance of social stimuli. In theory, such increased motivation towards social stimuli should increase the likelihood that people will encounter and engage with social opportunities, allowing them more opportunity to make up for lost social interactions. Identifying patterns such as these and assessing their relationship with affective outcomes can help us to better understand which kinds of behavioural responses are conducive (or otherwise) to building psychological resilience in response to reduced social interaction. Since automatic tendencies can be trained over time, interventions that encourage individuals to approach social stimuli, could then be used to protect vulnerable individuals against psychological decline.

Therefore, this research is important not only to help us understand more about the interrelationship between behaviour and approach-avoidance tendencies in general, but also to help governments worldwide build a clear picture of the extent to which policies that encourage reductions in social behaviour, may impact the psychological health of their citizens. It can also help to identity pathways through which to mitigate the negative impacts of reduced social interaction, and loneliness, more generally.

### The present work and theoretical framework

Only few studies so far investigated approach and avoidance in the context of the COVID-19 pandemic. In summary, it was found that individual differences in approach, relative to avoidance, were more important predictors of compliance with COVID-19 recommendations, and were positively associated with social distancing, wearing masks and gloves, and reduced mobility^17^. Moreover, individual differences in avoidance were associated with impaired wellbeing during the pandemic^18^, and self-reported mask-related worrying was associated with lower avoidance bias toward unmasked people, but only for participants with low COVID-19 anxiety^19^. However, our study is the first to investigate whether changes in isolated, in addition to more general, social behaviours are associated with changes in approach-avoidance tendencies.

To examine this, we measured adherence to some of the main restrictions on social behaviours set out by the UK government (see Table 1) as well as approach-avoidance tendencies in response to sad/happy faces and social scenes at three different time points over three months. We gathered these data during a period of lockdown easing in the UK (May–July 2020) following the country’s first strict lockdown in March (see “Methods” Table 7 for more detail of lockdown context across waves). All data were gathered using online surveys and approach-avoidance was measured using the Stimulus Response Compatibility Task, which required participants to press a computer key to make a manikin on the screen move towards (approach) or away from (avoid) the picture.

**Table 1:** Social distancing behaviours measured.

1. Social distancing item	I have been social-distancing (keeping 2 m apart from other people outside of the house)
2. Self-isolating item	I have been self-isolating (i.e. not being in contact with anyone else or leaving the house)
3. Avoiding crowds item	I have been avoiding crowds
4. Avoiding small groups item	I have been avoiding small group face-to-face activities with friends and family from outside of my house
5. Reduced in-person interaction item	I am having less in-person social interaction than before social distancing measures were first introduced
6. Reduced-overall-interaction item	I am having less overall social interaction (digital or in person) than before social distancing measures were introduced
7. Mask outdoors item	I have been wearing a mask outdoors

There are two main theoretical accounts that can inform our predictions: compatibility hypothesis^20^ and emotion regulation theory^21^. According to the compatibility hypothesis that has appeared in various formulations across many articles^4,22–27^ negative (positive) affect, emotions, and experiences are compatible with avoidance (approach) motivation and should thus facilitate avoidance (approach) responses. Therefore, avoidance (approach) can be conceptualized as a preparedness to respond to negative (positive) objects^27^. For example, people are faster to push rather than pull various negatively valenced stimuli, from negative words^28^ to images of spiders they are afraid of^29^. These positive/negative stimulus evaluations are usually grounded in innate or learned tendencies^30^. In that regard, we might expect a link between reduced social behaviours and reactions to different faces that complies with an extended version of the compatibility hypothesis. For example, reduced social interaction may be an inherently negative experience given its adverse consequences for mental health^2,31–33^ and thus further potentiate avoidance responses to the compatible negatively valenced faces. In other words, if reduced social behaviours are negatively valenced experiences, they may simply predispose people to react more quickly to avoid sad faces as they are emotionally congruent stimuli.

Emotion regulation theory broadly refers to people’s attempts to influence emotions in themselves and others^21,34,35^. There are five families of emotion regulation strategies, and two of them are particularly relevant in the context of the present research: situation modification and attentional deployment^21^. Situation modification involves undertaking action to change a given situation in order to experience desired emotions, whereas attentional deployment involves directing attention away from (or toward) stimuli that evoke undesirable (desirable) emotions. Therefore, it is plausible that people may undertake approach-avoidance reactions regarding images of faces, and this could fall both under situation modification (i.e., changing the situation by pushing or pulling specific faces) and attentional deployment (i.e., pulling certain faces toward oneself or pushing them away to direct attention toward or away from these faces).

In that regard, an argument could be made that, if people naturally associate reduced social behaviours with feeling negative, they may experience an inclination to approach (vs. avoid) happy faces and avoid (vs. approach) sad faces. The idea being that this inclination functions to protect them against the potential for experiencing negative feelings in the future. Therefore, the present work focused on testing for a direct association between changes in social behaviours with changes in approach-avoidance responses. Individuals may also have stronger inclinations to approach (vs. avoid) social stimuli more generally, given that they may perceive assuaging the need for social interaction by exposing themselves to social stimuli as a response that will bring about positive feelings. Similar self-regulating feedback loops between approach-avoidance tendencies and affect have been proposed by prominent approach-avoidance theorists and thus warrant testing in a natural setting^36^.

Overall, both the compatibility hypothesis^20^ and emotion regulation theory^21^ imply that approach-avoidance tendencies can be meaningfully shaped by negative experiences. Since many studies have shown that reducing social behaviour is an inherently negative experience we predict that reduced social behaviour will shape approach-avoidance tendences toward different social stimuli. However, the two theories yield different predictions. As discussed, the emotion regulation theory predicts that reduced social behaviours will be linked to both stronger approach relative to avoidance tendencies toward happy faces (**H1**), and to stronger avoidance relative to approach tendencies regarding sad faces (**H2**), as a strategy to maintain a more optimal emotional state. Similarly, the theory predicts that reduced social behaviours will be linked to stronger approach relative to avoidance tendencies toward social stimuli (**H3**), given that these behavioural reactions may serve to activate desired motivational states by assuaging the need for social interaction. In contrast, the compatibility hypothesis predicts only **H2**, given the compatibility of negatively valenced sad faces with avoidance tendencies and reduced social behaviours that are inherently negative.

## Statistical approach

The aim of the analysis was to explore whether changes in social behaviours are significantly associated with changes in approach and avoidance behaviour over time. Since the nature of this study was exploratory, we began by first checking descriptive statistics for all input and outcome variables. We then ran three balanced, fixed effects panel linear models on the respective approach-avoidance outcomes of interest (happy, sad, and social tendency) using the 7 prescribed social behaviours as predictors to assess whether any significant relationships were present. Panel linear regression was chosen since it is free from distributional assumptions^37^. Each of the four tendencies was computed as the difference between the participant's mean response time to start a correct manikin movement away from the corresponding pictures (avoidance) minus the mean response time to start a correct movement towards the pictures (approach). For instance, the sad tendency reported below was computed as: Mean RT to start a movement away from sad faces minus mean RT to start a movement towards sad faces. Positive values of these tendency scores reflect relative approach of the stimulus category, whereas negative values reflect relative avoidance. In previous studies, typical ranges of these scores were between − 50 ms and + 50 ms.

To account for multiple testing, we applied the False Discovery Rate Controlling Procedure^38^ to all reported p-values below. Additionally, to account for possible multicollinearity issues arising from correlations between the social behaviours (despite the fact that these correlations were not decidedly large^39^, Appendix Table 1), we ran separate models for each of the 3 outcomes using each single behaviour as a predictor on its own to determine whether this changed any results from significant to insignificant or vice versa. To test whether affective variables—or any other of the measured time-varying variables—were partially responsible for any observed effect, we also reran the singular behavioural predictor model with all additional time-varying covariates measured in the study.

The covariate model included several key affective variables, including anxious, happy, and stressed today, valence, arousal, social anxiety, anxiety, and life satisfaction. These variables were included to control for their potential influence on the relationship between reduced overall interaction and sad tendency. For example, reduced-overall-interaction might only affect sad tendency because of its impact on negative affectivity. If this were the case, then removing variables associated with negative affectivity should alter the strength of the association between reduced-overall-interaction and sad tendency. Thus, including affective variables helps us to partition out the unique variance associated with these variables.

Fear of contracting Covid-19 both for oneself and for others were included to account for the potential impact of the Covid-19 context on the relationship between reduced overall interaction and these tendencies. For example, it might be that reduced overall interaction is highly associated with fear of contracting Covid-19, and it is this fear rather than the reduced interaction itself that impacts upon sad tendency. In addition, Behavioural Inhibition System (BIS—corresponds to motivation to avoid aversive outcomes) and Behavioural Approach System (BAS—corresponds to motivation to approach goal-orientated outcomes)) tendencies were included to control for people’s general sensitivity towards positive and negative stimuli^40^ and especially since they have been previously associated with infection avoidance^41^.

Since there was no obvious reason for us to favour a fixed over a random effects model based on our data structure, we ran both covariate models and conducted the Hausman test to compare model fit^42^. We also ran a random effects plm with non-time varying variables (age, gender, ethnicity, income, region, mental health, physical health, subjective wellbeing, Big 5 personality traits, number of children, number of people living in the house, keyworker status and covid-19 symptoms) since inclusion of time non-varying variables was not possible with fixed effects plms. This helped to account for the possible impact of individual differences on any observed association between social behaviour and approach-avoidance and partial out any variance associated with these more general factors.

To improve generalisability and understanding of these data, the covariate models listed above were conducted on three dataset variations: (1) balanced panel dataset including only participants with data at all three time points, (2) maximum sample dataset that included participants with data from 2 time points or 3 time points, (3) maximum change dataset that included only participants with data from time points 1 and 3, where the largest changes in social behaviour had taken place.

To account for the possibility that it is not the social behaviours themselves but rather participants conscious feelings (e.g. conscious feelings regarding their day yesterday, how they feel right now, or more generally) that may be associated with these behaviours driving any observed effect, we also tested whether negative affective variables were significantly associated with social behaviours in separate fixed effects panel linear models, for instances where a significant association between social behaviour and approach-avoidance was found. Finally, to obtain a more complete understanding of the interrelationships between social behaviours, negative affectivity, and approach-avoidance, we tested for an association between our measured affective variables with the relevant approach-avoidance tendency, also using panel linear models (e.g. anxious today predicting sad tendency).

## Results

### Descriptive statistics

Below we report the descriptive statistics for sad tendency and social behaviour 6: reduced overall (digital and in person) interaction. We report these variables since, as can be seen in section “Fixed effects panel linear model results” below, a statistically significant relation was found between them, and they are thus the central focus of this paper. Descriptive statistics and further analyses of the other tendency outcomes are reported in the appendix (Tables A. 2–8).

As can be seen in Table 2, people became more avoiding of sad images on average in wave 2 as compared to waves 1 and 3. However, a repeated measures ANOVA with time as the independent variable and sad tendency as the dependent variable indicated that these changes were not significant (F(2,346) = 1.161, p = 0.314).

**Table 2:** Sad tendency means across waves.

Wave	Mean	SD	Count
1	6.102	324.141	174
2	− 36.771	302.293	174
3	2.292	285.294	174

As per Table 3, people reported lower values for reduced-overall-interaction on average over time, meaning that as time went on and lockdown restrictions eased, people interacted more. However, a repeated measures ANOVA with time as the independent variable and reduced-overall-interaction *overall* as the dependent variable indicated that these changes were not significant F(2,346) = 1.782, p = 0.17).

**Table 3:** Reduced-overall-interaction means across waves.

Wave	Mean	SD	Count
1	7.023	3.149	174
2	6.665	3.151	174
3	6.483	3.147	174

### Fixed effects panel linear model results

In the fixed effects panel linear model exploring the impact of the 7 behavioural predictors on sad tendency, we found that reduced-overall-interaction was a significant predictor of sad tendency (tendency to approach over avoid sad faces, see Table 4). Specifically, it was found that reduced overall interaction was significantly associated with stronger avoidance relative to approach of sad faces over time. With each one-unit change in reduced-overall-interaction (measured on a 0–11 scale), sad tendency reduced by about 18 ms. This relation remained significant following FDR correction. It also remained significant when ran as fixed effects model with less overall interaction included as the only predictor variable (Table A.9), and when ran as a random effects panel linear model (Table A.10).

**Table 4:** Simple fixed effects panel linear model showing a significant association between reduced-overall-interaction and sad tendency.

	Estimate	Std. Error	P
Social distancing	7.619	12.726	0.550
Self-isolating	-8.906	6.316	0.159
Avoiding crowds	12.413	13.317	0.352
Avoiding small groups	-2.674	7.562	0.724
Reduced *in person* interaction	-0.226	8.529	0.979
Reduced *overall* interaction	-17.811	6.426	0.006**
Mask outdoors	2.645	6.057	0.663
N	174		
F	1.922		
R^2^	0.038		
Cohen’s *d* for reduced *overall* interaction	0.210		

Note: Fixed effects regression using prescribed social behaviours (independent variables) to predict sad tendency (dependent variable). Standard errors are clustered on an individual level. Cohen’s *d* value was calculated using the reduced-overall-interaction coefficient to estimate effect size. * p < 0.05, ** p < 0.01, *** p < 0.001.

Overall, this result was consistent with the predictions made from the compatibility hypothesis (**H2**: reduced social behaviours will be linked to stronger avoidance relative to approach tendencies regarding sad (but not happy or social faces) given the affective compatibility.

In all three fixed effects covariate panel linear models, the significant association between reduced-overall-interaction and sad tendency remained (Table 5). The strongest model was model 3 which was conducted on the maximum change dataset (R^2^ = 0.139). In this model, a one unit increase in reduced-overall-interaction (0–11 scale) was associated with a 26.555 ms decrease in sad tendency (tendency to approach over avoid sad faces). Therefore, reduced overall interaction was associated with higher avoidance over approach of sad stimuli.

**Table 5:** Reduced-overall-interaction is significantly associated with sad tendency when all time-varying variables are included in the fixed effects panel linear model.

	Dependent variable:		
	Sad tendency		
	Balanced	Maximum sample	Maximum change
Reduced-overall-interaction	− 18.733***	− 17.281***	− 26.555***
	(5.713)	(5.483)	(7.592)
Corona fear	10.922	10.193	**− **11.547
	(15.246)	(13.993)	(18.601)
Corona fear others	**− **8.934	**− **7.989	9.447
	(14.202)	(12.862)	(18.129)
Anxious today	4.063	5.689	25.528
	(12.147)	(11.416)	(16.431)
Happy today	**− **25.855*	**− **26.631*	**− **2.842
	(15.491)	(14.857)	(22.782)
BIS	4.940	2.284	8.617
	(7.190)	(6.963)	(8.773)
BAS Drive	6.650	6.279	4.002
	(10.061)	(9.482)	(12.493)
BAS Reward	5.582	4.856	1.384
	(5.894)	(5.486)	(6.513)
Hours away from home	**− **0.057	**− **1.974	14.447
	(15.622)	(14.847)	(20.236)
Stressed today	**− **25.596**	**− **28.009**	**− **39.730**
	(12.246)	(11.575)	(15.626)
Social Anxiety	**− **0.536	**− **0.887	**− **1.267
	(1.019)	(0.967)	(1.342)
Anxiety	**− **8.998**	**− **4.926	**− **11.900**
	(3.960)	(3.684)	(5.486)
Life Satisfaction	11.111	13.994	17.739
	(21.277)	(20.394)	(29.361)
Valence	0.530	0.206	**− **0.956
	(1.179)	(1.069)	(1.466)
Arousal	**− **1.265	**− **1.098	**− **1.040
	(0.896)	(0.851)	(1.188)
N	522	608	415
R^2^	0.075	0.059	0.139
Adjusted R^2^	**− **0.456	**− **0.548	**− **1.015
F Statistic	1.784** (df = 15; 331)	1.542* (df = 15; 369)	1.899** (df = 15; 177)
Cohen’s *d* for less overall interaction	**− **0.144	**− **0.138	-0.153

Note: ^*^p < 0.1; ^**^p < 0.05; ^***^p < 0.01.

Fixed effects regression using prescribed social behaviours (independent variables) and other time varying covariates to predict sad tendency (dependent variable). Standard errors in brackets are clustered on an individual level. Cohen’s *d* value was caculated using the reduced-overall-interaction coefficient to estimate effect size.

### Random effects panel linear model results

In the random effects covariate panel linear model including additional time-varying predictors (Table 6), the significant impact of reduced-overall-interaction on sad tendency also remained. This significant association also remained when including time non-varying variables into the random effects model (Table A.11).

**Table 6:** Reduced-overall-interaction is significantly associated with sad tendency when all time-varying variables are included in the random effects panel linear model.

	Dependent variable:		
	Sad tendency		
	(1)	(2)	(3)
Reduced-overall-interaction	**− **15.599***	**− **12.517***	**− **15.828***
	(4.509)	(4.216)	(5.095)
Corona fear (self)	1.872	1.915	**− **1.850
	(7.830)	(6.961)	(8.544)
Corona fear (others)	**− **1.298	**− **1.657	2.621
	(8.437)	(7.419)	(9.235)
BIS	**− **4.577	**− **5.955	**− **7.168
	(4.660)	(4.243)	(4.975)
BAS Drive	7.539	4.023	**− **2.283
	(6.351)	(5.847)	(7.074)
BAS Reward	**− **1.160	0.268	**− **0.671
	(3.330)	(3.152)	(3.465)
Stressed today	**− **10.190	**− **11.451	**− **15.544
	(9.353)	(8.709)	(10.386)
Anxious today	12.143	11.047	18.442*
	(9.039)	(8.341)	(10.206)
Happy today	**− **14.824	**− **15.896	**− **12.072
	(11.968)	(11.210)	(14.406)
Social Anxiety	**− **0.054	**− **0.271	**− **0.585
	(0.563)	(0.518)	(0.636)
Anxiety	**− **0.146	1.713	1.069
	(2.278)	(2.080)	(2.537)
Life Satisfaction	6.249	10.418	16.028
	(14.359)	(13.149)	(16.502)
Valence	0.972	0.692	**− **0.401
	(0.904)	(0.816)	(0.984)
Arousal	**− **0.970	**− **0.720	**− **0.304
	(0.615)	(0.567)	(0.710)
Hours away from home	14.044	11.595	12.302
	(11.732)	(10.747)	(12.971)
Constant	144.826	106.980	227.751
	(194.515)	(179.019)	(216.393)
Observations	522	598	415
R^2^	0.040	0.028	0.045
Adjusted R^2^	0.011	0.003	0.010
F Statistic	20.816	16.999	18.974
Cohen’s d for reduced-overall-interaction	**− **0.151	**− **0.130	**− **0.134

Note: ^*^p < 0.1; ^**^p < 0.05; ^***^p < 0.01.

Notes: Random effects panel linear regression using reduced-overall-interaction and all other time varying covariates (independent variables) to predict sad tendency (dependent variable). Standard errors for each estimate are listed in brackets. Cohen’s d value was caculated using the reduced-overall-interaction coefficient to estimate effect size.

The Hausman test revealed that neither model produced significantly different results (p = 0.163), therefore the random effects model was chosen as the best specification for our data.

### Relationships between affective variables with reduced-overall-interaction and sad tendency

Counter to expectations, none of the affective variables were significantly associated with reduced-overall-interaction in our fixed effects panel linear models (Tables A.12–14).

In the fixed effects panel linear models exploring whether changes in affective variables were associated with changes in sad tendency, we did find some significant effects (Tables A.15–17). Increases in generalised anxiety levels were associated with less approach over avoidance of sad faces (β = − 8.703**). Therefore, the more anxiety people experienced over time, the faster they were to avoid (relative to approach) sad faces. We also found a significant negative association between stressed today and approach over avoidance of sad faces (sad tendency—β = − 19.030**). Therefore, higher levels of stress were associated with less approach (relative to avoidance) of sad images. We note that these significant associations were not present for happy or social tendency, only sad tendency. Neither fear of infecting oneself nor of infecting others with the corona virus was associated with sad tendency.

## Discussion

COVID-19 facilitated an unprecedented shift in social behaviour across the globe. This study used the prescribed changes in behaviour set out by the UK COVID-19 policy guidelines as a natural setting to assess whether changes in social behaviours are associated with changes in approach-avoidance tendencies over time. To this end, during a period of lockdown and easing of social restrictions, we asked people about the extent to which they had been following several socially relevant policy guidelines over the last 7 days. We also measured their approach-avoidance tendencies in response to sad/happy faces, and social scenes, at three time points, each one month apart. Research of this kind can provide the best possible evidence for relevant real-world phenomena when random assignment of participants to experimental conditions is not possible. We found a significant relationship between “less overall (digital and in person) social interaction since social distancing measures were first introduced” and participants’ tendency to approach over avoid sad faces (sad tendency). Specifically, reducing overall social interaction was related to more avoidance relative to approach of sad faces. The other prescribed social behaviours (social distancing, self-isolating, avoiding crowds, avoiding small groups, less in person interaction and wearing a mask outdoors) had no significant relationship with approach-avoidance tendencies.

Up until now, studies demonstrating the impact of real-world behaviour changes on approach-avoidance tendencies have been lacking. The relationship between behaviour and approach-avoidance has typically been shown in the inverse direction, whereby training people to alter their automatic approach-avoidance tendencies succeeds in altering their real-world behaviour^43–45^. This lack of research is likely because implementing and capturing widespread behaviour change over time can be challenging. To our knowledge, this research is the first of its kind to document a relationship between widespread behaviour and automatic approach-avoidance change in a natural setting. According to our most effective model (R^2^ = 0.139), with each one-unit change in reduced-overall-social-interaction (measured on a 0–11 scale), sad tendency reduced by about 26 ms. Whilst this effect size is small-medium^46^ (Cohen’s d = 0.153) the magnitude of this change is generally understood to be meaningful in the approach-avoidance literature^9^. Moreover, the magnitude is similar to the one frequently achieved by direct attempts to modify approach-avoidance tendencies, suggesting that continued investigations of the relation between real-world behaviour change and changes in automatic approach-avoidance tendencies are worthwhile.

Importantly, the negative association between reduced overall interaction and sad tendency remained robust to three different dataset variations as well as different model calculation methods (fixed and random effects). It also survived FDR correction; therefore, we believe the identified effect is a robust one. Reduced overall interaction was most strongly associated with sad-face avoidance in the models that included other time-varying predictors, relative to the models that only included the 7 behavioural predictors. This is likely because many of the prescribed social behaviours captured will have shared some variance with one another. Therefore, we considered the covariate model results to be a more accurate and robust representation of impact. However, the model including all 7 prescribed social behaviours was important to determine whether any of the behaviours in question were significantly associated with approach-avoidance tendencies over and above the other behaviours, which was the case for reduced-overall-interaction.

We consider the possible impact of reduced overall interaction on sad-face avoidance (and not happy face or social scene avoidance) to be consistent with the compatibility hypothesis insofar as our Hypothesis 2 was supported. If people were adapting their automatic approach-avoidance tendencies to regulate their negative affectivity in response to reduced social behaviours as per emotion regulation theory, then we would have expected to see an association between reduced social behaviours with happy and social, in addition to sad, tendencies. We would also have expected to see an association between reduced-overall-interaction and the affective variables, which we did not. Instead, our results appear to be a better fit with the compatibility hypothesis, which contends that people respond to affective experiences with compatible approach-avoidance responses. For example, somebody feeling negative should be faster to respond with avoidance to negative images. We specifically predicted therefore, that since reduced social interaction is an inherently negative experience for humans to undertake, it would facilitate faster compatible responses to negative images (e.g., faster avoidance relative to approach of sad faces) (H2).

Since reduced-overall-interaction was not associated with affect self-reports, it is possible that the link between reduced-overall-interaction and increased avoidance relative to approach is direct and cannot be explained by conscious affective experiences. It may be the case then that our automatic approach-avoidance based response system causes an immediate response which serves to protect us against downstream negative effects of extreme isolation before they have taken hold. Our research is therefore aligned with the prospect that automatic approach-avoidance responses function as a precursor to affective responses (in addition to being driven by them).

Consistent with more general findings, we did find an important association between changes in affect and changes in approach-avoidance over time. However, to our knowledge, this is the first time that such associations have been demonstrated in a natural setting. Namely, we found significant negative associations between changes in generalised anxiety over time, and changes in momentary stress levels over time, with sad tendency. That is, increases in general anxiety and stress yesterday were associated with decreases in people’s tendency to approach over avoid sad images. This latter finding is also consistent with the compatibility hypothesis since we observe an association between negative affective traits/states with compatible avoidance responses to negative stimuli (sad faces) and not positive or social stimuli (happy faces and social scenes)^47^. This avoidance of negative stimuli in response to negative affective states/traits may function in a similar way to the processes observed in optimism bias research. This research find that healthy individuals show a bias towards more desirable stimuli which is thought to enable healthy psychological functioning^48^. Since faster avoidance relative to approach of aversive stimuli is generally considered adaptive, this tendency might have again increased to bolster individuals against downstream reductions in mental wellbeing^49^.

Importantly, reductions in in-person interaction alone were not significantly associated with sad tendency, rather it was only the combination of both digital and in person interactions (reduced-overall-interaction) that had an impact. Presumably, circumstances where people substitute in person for digital interactions may negate the need for a mechanism to reduce any negative affect that arises from that loss of interaction. Indeed, research has shown that digital interaction can buffer against the negative psychological impacts of social isolation^50^, perhaps due to its ability to function as an alternative social outlet^51^. This highlights the potential importance of online platforms that enable communication during periods of social restrictions. It also suggests that those unable to shift their social interactions to an online format may be at increased risk from policies that restrict face-to-face interactions and thus ought to be prioritised by government interventions seeking to mitigate the negative impact of the pandemic on the public. This substitution of reduced in person social interaction with online interactions can likely also explain the null impact of the other social behaviours on approach-avoidance tendencies. We recommend that future work seeks to disentangle the effects of online compensation for reduced social interaction in an experimental setting.

There are some notable limitations to this study. First, this study cannot prove a causal link between reduced-overall-interaction and sad tendency since behavioural and approach-avoidance variables were collected at the same three time points rather than one after the other. Whilst panel linear regressions are proficient at controlling for time invariant unobserved variables^52^, they cannot control for time varying omitted variables. The potential for this bias is less in panel data than it is in cross-sectional data. Nevertheless, it is possible that some unobserved variables or external events might explain the association. Second, in the present study, we focused on reducing type I error (i.e., false positive findings) by implementing FDR^53^ corrections, given that this error is responsible for the replication crisis in psychology^54^. However, we acknowledge that our emphasis on type I error may have inflated type II error^55^. In other words, we may have potentially failed to detect certain “real” effects because the corrections we used raised the bar for detecting an effect. Therefore, the stringent approach that we used may have prevented us from detecting certain smaller effects that could have potentially provided more nuanced insights into the link between reduced social behaviours and approach-avoidance tendencies. Moreover, since we did not see significant changes in reduced-overall-interaction over time, it is likely that the effect we observed may be underestimated, and therefore greater under conditions where significant changes are present. This is consistent with our finding that the association between reduced-overall-interaction and sad tendency becomes stronger when considering responses that were further apart in time. In terms of generalisability, it is possible that self-reported behaviours may not correspond perfectly to actual behaviours. Additionally, though we ran many tests to determine the robustness of the identified association between changes in social interaction with changes in sad tendency, it is important to note that most of the other social behaviour associations did not reach statistical significance. Thus, it is strongly recommended that the identified association is retested in future work. Finally, the necessity for a rapid response to an evolving situation meant it was not possible to conduct pre-validation studies on the social scenes used in this study. Therefore, future work should also seek to validate the social scenes presented since insufficient stimuli selection might have contributed to null results for these stimuli. 

Overall, this study has revealed a potentially important relationship between deliberative avoidance of social behaviours and automatic approach-avoidance tendencies. Namely, during the COVID-19 pandemic people appear to have responded to reduced social interactions by increasing the extent to which they avoided (relative to approached) sad stimuli. This underscores the potential adaptive significance of approach-avoidance tendencies in response to behaviour, rather than just as determinants of behaviour. Existing research on differences in approach-avoidance tendencies has focused either on stable differences caused by stimulus types (e.g., pleasant vs. unpleasant stimuli such as spiders vs. butterflies) or on stable inter-individual differences (e.g., spider fearful vs. non-fearful, patients vs. healthy controls). The current study shows that is also worthwhile to investigate temporal changes and intra-individual differences.

It is highly possible that such findings, although observed during the COVID-19 pandemic, may also refer to isolation and reduced social interaction more broadly. Policymakers may use these data as a starting point to better understand the potential impacts of social isolation, and identify individuals who may be at increased risk of declining wellbeing in response (e.g. those who are unable to compensate in person interaction with digital interaction). Researchers can use these data as a foundation from which to design new experiments and interventions that test the causality of these associations, for example, by seeing whether training avoidance of sad stimuli can help to increase wellbeing in the face of loneliness. If successful, policymakers can administer such trainings to members of the public at high risk of social isolation. As such, we encourage researchers interested in behaviour, wellbeing, and motivation, to embark on a new line of research that further explores the role of approach-avoidance tendencies as adaptive responses to changes in wellbeing-related behaviours.

## Methods

### Data collection

This study ran between 23rd May and 20th August 2020. We sampled from a nationally representative online population in the UK at three time points over three months. Whilst the study was UK based, the social behaviours investigated were consistent with those put in place by many other countries across the globe. A month between waves was selected as time a period over which changes in social behaviour were expected to have taken place due to the easing of lockdown restrictions. The dates and lockdown contexts for each of the three waves is detailed in Table 7 below. Participants were recruited via PureProfile online study recruitment agency and received £4 for participating in the study. All methods were performed in accordance with the relevant guidelines and regulations. The experimental protocol was approved by the London School of Economics Ethical Committee (ref#1133). Informed consent was obtained from all participants.

**Table 7:** Dates of each wave and associated UK COVID-19 restriction context^56^.

	Date	Lockdown status
Wave 1	23rd May 2020	First lockdown has started to ease, people are allowed to leave the house to sunbathe and exercise more than once a day. People must keep two metres away from others and are also encouraged to wear face coverings in enclosed places. It is not possible to meet others in groups, any schools are closed, and non-essential shops are shut
Wave 2	22nd June 2020	Virus alert level downgraded from four to three. Schools have gone back. Non-essential shops are back open. People are now allowed to meet outside in groups of up to 6
Wave 3	21st July 2020	Work-from-home guidance eased as England plans for return to normality. Pubs are open again and weddings allowed. On 24^th^ July face covering becomes mandatory

### Participants

There were 1097, 325 and 267 consenting participants obtained in each wave. From this pool, we removed those who: 1) did not have both survey and approach avoidance data, 2) had two or more responses for one wave, and 3) took less than 600 s (10 min) to complete the survey. This cut of point was decided based on it being close to the lowest in the distribution of response durations. This left us with a remaining sample of 364, 259 and 224. Attrition was due to difficulties with downloading the software required to play the approach-avoidance task online. Those who dropped out of the study and those that stayed in were comparable across gender, age, and income (Appendix, Tables 22–24). Finally, since we were interested in changes within people over time, we removed those that did not have data in all three waves, as well as cases with missing approach-avoidance data, which were not feasible for analysis. A final sample of 174 remained in each wave. The present research was well powered to detect at least medium effect sizes, regardless of the alpha level used (for full calculation description see Appendix, sample size calculation, page 15).

Out of the 174 participants in this study, 53% were male and 47% were female; 20% were key workers, 80% were not. There was a roughly even split across age groups (14–30% each in age brackets 25–34, 35–44, 45–54, 55–64 and 65 +) although there was less representation for ages 18–24 (2%). The sample had good representation across income levels and regions. However, it lacked representation from other ethnic groups since 97% of the sample were white, which we address as a limitation of the study (see Appendix, Tables 18–21 for full breakdown).

### Procedure

We administered an online survey to the same people at three different time points spaced one month apart. The online survey consisted of various survey questions and a Stimulus Response Compatibility Task (SRCT; also called “manikin task”), which measures automatic approach-avoidance tendencies. The survey questions included items on mood, wellbeing, personal traits, and adherence to 7 of the prescribed social behaviours. After answering these questions, participants completed the SRCT. During the SRCT, participants were presented with an image in the centre of the screen, and a matchstick man stood either above or below the image. Participants had to pay attention to whether the image was tilted to the right or left. They were told that hitting the B key made the man run down and hitting the Y key made the man run up. If the image was right-tilted, they were instructed to make the matchstick man AVOID the image by hitting the correct key (B or Y, depending on whether the man was located below or above the image). If the image was left-tilted, they were instructed to make the matchstick man APPROACH the image, using the same keys. When the B or Y key was pressed, a short movie was started, showing the manikin move slightly downwards or upwards, respectively. The task contained images of social scenes, as well as positive and negative facial expressions. We used participant button press reaction times to measure approach-avoidance tendencies. Full task description can be seen in Appendix, Fig. 1.

The happy and sad face images were sourced from the Radboud Faces Database. All face images had been rated by a large pool of diverse participants according to their valence and arousal dimensions^57^. We selected 8 male and 8 female actors from the Radboud Faces Database; each one expressed each emotion, yielding 16 positive (happy) facial expressions and 16 negative (sad) ones. In addition, 32 social scene images were sourced from three databases of images^58–60^, which are widely used by the academic community. Social images were diversified across image sub-types: crowds, everyday scenes, pairs, and small groups. Each participant first completed 16 practice trials, then 60 happy/sad face image trials, then 20 social image trials, yielding a total duration of approx. 10 min. For each of the 4 image types, 10 pictures had to be approached and 10 had to be avoided by the manikin. Across the 4 image types, we counterbalanced which pictures had to be approached versus avoided. From the available pictures, the program randomly picked the ones to be used for each test run.

Since it was not possible to seamlessly integrate the SCRT into the survey form, to complete the task participants were directed to a separate online link which required a software download to run the task. To match the SCRT data with the survey data, participants were required to enter a memorable word both in the survey form and on the task link before completing the SCRT. A separate file was then created containing data where we were able to achieve a match between survey and online task data and a separate file was created with these participants only for data analysis.

### Measures

We began the survey by asking participants to report their subjective wellbeing based on the four questions used by the Office for National Statistics in the UK: “*Overall, how satisfied are you with your life nowadays?*” measuring the evaluative dimension; “*Overall, how worthwhile are the things that you do in your life?*” measuring the eudemonic dimension; “*Overall, how happy did you feel yesterday?*” and “*Overall, how anxious did you feel yesterday?*” both measuring the affective dimension. To capture stress we added the question, “*Overall, how stressed did you feel yesterday?”.* Reports are on a 0–11 scale, from ‘not at all’ to ‘completely’.

We then measured affect using the Affective Slider^61^ which allows for the relative assessment of valence and arousal on a comparative scale. This is important when trying to determine which properties of moods might be linked to different motivational tendencies.

This was followed by two questions where participants had to indicate the extent to which they agreed with the following statements: “*I am worried about catching the Coronavirus (for me)”* and “*I am worried about catching the Coronavirus (for others)”.*

We then measured adherence to prescribed social behaviours (Table 1) by asking participants to indicate “the extent to which the following statements describe your behaviour over the past 7 days” on a scale of 0 (not at all) to 11 (completely).

This was followed by several self-report trait measures including the State Trait Anxiety Inventory^62^, the Liebowitz Social Anxiety Scale^63^, a brief measure of personality (Big 5)^64^ and the BIS/BAS scale^40^ which is designed to measure two motivational systems: the behavioural inhibition system (BIS) and the behavioural activation system (BAS), which correspond to approach-avoidance tendencies. Finally, we asked a series of questions about participants’ current circumstances (e.g. COVID-19 symptoms, how many people they live with, and general health) as well as demographics. After filling out this online survey, participants completed the SCRT.

### Model descriptions

Fixed effects were selected as primary models for their ability to omit unobserved variable bias. However, random effects were also conducted on significant outcomes for comparison and robustness. Since we were interested in whether changes in behaviour were significantly associated with changes in approach-avoidance tendencies within people over time, we mainly focused on participants with data points on these variables for all three waves. However, for robustness, and to deepen our understanding of the data, we also ran models on a maximum power sample, which included people with data from 2 time points in addition to those with 3 time points, and a maximum change sample which included people with data from time points 1 and 3 only, where we will have seen the biggest change in social behaviour over time. Tendency scores were calculated by subtracting the mean approach reaction times from the mean avoidance reaction times for each stimulus type: happy, sad, and social.

In all initial panel linear models, social behaviours 1–7 were entered as predictors and ID and Time were kept constant. Any identified effects of social behaviour on approach-avoidance tendency outcomes from these models were then explored further in more robust panel linear models which included all time-varying covariates.

An example fixed effects model we tested to explore the relationship between less overall interaction and sad tendency is listed below.

$$\begin{aligned} Sad Tendency _{it} =& \mu _{t} + \beta_{1} behaviour6_{it} + \beta_{2} Corona \;fear \left( {self} \right)_{it} \\ &\; + \beta_{3} Corona\; fear \left( {others} \right)_{it} + \beta_{4} Anxiety_{it} + \beta_{5} Social \;Anxiety_{it} \\ & \; + \beta_{6} Anxious_{it} + \beta_{7} Happy_{it} + \beta_{8} BIS_{it} + \beta_{9} BAS \;Drive_{it} \\ & \; + \beta_{10} BAS \;Reward_{it} + \beta_{11} Hours \;Away_{it} + \beta_{12} Stressed_{it} + \beta_{13} Life \;Satisfaction_{it} \\ & \; + \beta_{14} Valence_{it} + \beta_{15} Arousal_{it} + \alpha _{i} + \varepsilon_{it} ,\\ \end{aligned}$$

where μ_t_ is the intercept term which varies across time but not cases, α_i_ captures the entity effects (or fixed effects) for each individual participant i, t denotes every time (wave) for each participant and ε_it_ is the error term. A two-way analysis was chosen since we were interested in both subject and time effects. All models were run using the plm (panel linear regression)^65^ package in R Studio (Version 1.4.1717) and standard errors were clustered at the level of individual i in all models.

Using the overall sample standard deviation of the Behaviour 6 (less-overall-social-interaction) coefficient, we computed Cohen’s *d* to assess the magnitude of the significant effects. Data and statistical analyses are available on request.

## Supplementary Information

Supplementary Information.

## Data Availability

The datasets generated and analysed during the current study are available in the The Open Science Framework repository, [https://osf.io/ydqgm/].
