# Supplementary material for: Pandemic related changes in social interaction are associated with changes in automatic approach-avoidance behaviour
Source: Sci Rep. 2023 Mar 21;13:4637. doi: 10.1038/s41598-023-31447-5 (PMC10029793; doi:10.1038/s41598-023-31447-5)
Supplement: Supplementary file 1 — Supplementary Information. [file 41598_2023_31447_MOESM1_ESM.docx]

**Appendix**

List of tables

A.1. Kendal correlations between all social behaviours were (mostly) significantly associated with each other but small in magnitude

|  |  |  |  |  |  |  |
| --- | --- | --- | --- | --- | --- | --- |
|  | **Social distancing** | **Self-isolating** | **Avoiding crowds** | **Avoiding small groups** | **Less in person interaction** | **Less overall interaction** |
| **Social distancing** |  |  |  |  |  |  |
| **Self-isolating** | 0.184*** |  |  |  |  |  |
| **Avoiding crowds** | 0.553*** | 0.176*** |  |  |  |  |
| **Avoiding small groups** | 0.454*** | 0.273*** | 0.489*** |  |  |  |
| **Less in person interaction** | 0.323*** | 0.0103** | 0.357*** |  |  |  |
| **Less overall interaction** | 0.177*** | 0.0150*** | 0.148*** | 0.183*** | 0.410*** |  |
| **Mask outdoors** | -0.025 | 0.161*** | -0.052 | 0.039 | 0.01 | 0.075* |

A.2. Social behaviours 1-7 means and standard deviations across waves

|  | **Social distancing** | **Self-isolating** | **Avoiding crowds** | **Avoiding small groups** | **Less in person interaction** | **Less overall interaction** | **Mask outdoors** |
| --- | --- | --- | --- | --- | --- | --- | --- |
| Time 1 | M=9.098 SD=1.481 | M=4.391 SD=4.046 | M=9.356 SD=1.290 | M=8.603 SD=2.224 | M=8.592 SD=2.438 | M=7.023 SD=3.149 | M=2.397 SD=3.590 |
| Time 2 | M=8.661 SD=1.826 | M=3.598 SD=3.860 | M=9.023 SD=1.703 | M=7.672 SD=2.907 | M=8.080 SD=2.646 | M=6.655 SD=3.151 | M=3.534 SD=3.952 |
| Time 3 | M=8.529 SD=1.685 | M=3.178 SD=3.842 | M=8.598 SD=2.112 | M=6.793 SD=3.285 | M=7.799 SD=2.803 | M=6.483 SD=3.147 | M=5.218 SD=3.968 |

Happy tendency descriptive statistics and associated panel linear model

A.3. Happy tendency means across waves

| **Wave** | **Mean** | **SD** | **Count** |
| --- | --- | --- | --- |
| 1 | 23.9 | 357 | 175 |
| 2 | 49.6 | 412 | 175 |
| 3 | 48.4 | 317 | 175 |

Happy tendency is relatively low in wave 1 and almost doubles in waves 2 and 3. However, a repeated measures ANOVA with time as the independent variable and happy tendency as the dependent variable confirmed that these changes were not significant (F(2,336) = 0.289, p = 0.741).

A.4. Simple panel linear model on happy tendency: fixed effects

|  | **Estimate** | **Std. Error** | **p** |
| --- | --- | --- | --- |
| Social distancing | 3.677 | 15.680 | 0.815 |
| Self-isolating | -10.860 | 7.829 | 0.166 |
| Avoiding crowds | -19.764 | 16.477 | 0.231 |
| Avoiding small groups | 15.818 | 9.352 | 0.092 |
| Less in person interaction | -15.463 | 10.532 | 0.143 |
| Less overall interaction | 4.757 | 7.915 | 0.548 |
| Mask outdoors | 4.730 | 7.507 | 0.529 |
| N | 175 |  |  |
| F | 1.177 |  |  |
| R^2^ | 0.024 |  |  |

Notes: Fixed effects regression using prescribed social behaviours (independent variables) to predict happy tendency (dependent variable). Standard errors are clustered on an individual level. * p<0.05, ** p<0.01, *** p<0.001

A.5. Simple panel linear models 1-7 on happy tendency, each model focusing on a single social behaviour in isolation: fixed effects

|  | | | | | | | |
| --- | --- | --- | --- | --- | --- | --- | --- |
|  | *Dependent variable:* | | | | | | |
|  |  | | | | | | |
|  | Happy tendency | | | | | | |
|  | (1) | (2) | (3) | (4) | (5) | (6) | (7) |
|  | | | | | | | |
| Social distancing | -2.773 |  |  |  |  |  |  |
|  | (14.604) |  |  |  |  |  |  |
|  |  |  |  |  |  |  |  |
| Self-isolating |  | -10.463 |  |  |  |  |  |
|  |  | (7.615) |  |  |  |  |  |
|  |  |  |  |  |  |  |  |
| Avoiding crowds |  |  | -18.188 |  |  |  |  |
|  |  |  | (14.873) |  |  |  |  |
|  |  |  |  |  |  |  |  |
| Avoiding small groups |  |  |  | 8.995 |  |  |  |
|  |  |  |  | (8.757) |  |  |  |
|  |  |  |  |  |  |  |  |
| Less in person interaction |  |  |  |  | -12.272 |  |  |
|  |  |  |  |  | (9.182) |  |  |
|  |  |  |  |  |  |  |  |
| Less overall interaction |  |  |  |  |  | -0.003 |  |
|  |  |  |  |  |  | (7.018) |  |
|  |  |  |  |  |  |  |  |
| Mask outdoors |  |  |  |  |  |  | 6.770 |
|  |  |  |  |  |  |  | (7.443) |
|  |  |  |  |  |  |  |  |
|  | | | | | | | |
| Observations | 525 | 525 | 525 | 525 | 525 | 525 | 525 |
| R^2^ | 0.000 | 0.005 | 0.004 | 0.003 | 0.005 | 0.000 | 0.002 |
| Adjusted R^2^ | -0.510 | -0.502 | -0.504 | -0.506 | -0.502 | -0.510 | -0.506 |
| F Statistic (df = 1; 347) | 0.036 | 1.888 | 1.495 | 1.055 | 1.786 | 0.000 | 0.827 |
|  | | | | | | | |

Notes: Fixed effects regression using each prescribed social behaviour (independent variable) to predict happy tendency (dependent variable) in separate models. Standard errors are clustered on an individual level. * p<0.05, ** p<0.01, *** p<0.001

Social tendency descriptive statistics and associated panel linear model

A.6. Social tendency means across waves

| **Wave** | **Mean** | **SD** | **Count** |
| --- | --- | --- | --- |
| 1 | 53.5 | 339 | 175 |
| 2 | 31.3 | 323 | 175 |
| 3 | 19.6 | 354 | 175 |

Social tendency is relatively high in wave 1 and reduces in waves 2 and 3. However, a repeated measures ANOVA with time as the independent variable and social tendency as the dependent variable confirmed that these changes were not significant (F(2,342) = 0.48, p = 0.619).

A.7. Simple panel linear model on social tendency: fixed effects

|  | **Estimate** | **Std. Error** | **p** |
| --- | --- | --- | --- |
| Social distancing | -12.8201 | 14.4748 | 0.3764 |
| Self-isolating | 4.0108 | 7.3191 | 0.5841 |
| Avoiding crowds | -5.6714 | 15.2022 | 0.7093 |
| Avoiding small groups | -7.7185 | 8.6102 | 0.3707 |
| Less in person interaction | 14.3128 | 9.7198 | 0.1418 |
| Less overall interaction | -3.4199 | 7.4078 | 0.6446 |
| Mask outdoors | 1.3941 | 6.9156 | 0.8404 |
| N | 175 |  |  |
| F | 0.604 |  |  |
| R^2^ | 0.012 |  |  |

Notes: Fixed effects regression using prescribed social behaviours (independent variables) to predict social tendency (dependent variable). Standard errors are clustered on an individual level.

* p<0.05, ** p<0.01, *** p<0.001

A.8. Simple panel linear models 1-7 on social tendency, each model focusing on a single social behaviour in isolation: fixed effects

|  | | | | | | | |
| --- | --- | --- | --- | --- | --- | --- | --- |
|  | *Dependent variable:* | | | | | | |
|  |  | | | | | | |
|  | Social tendency | | | | | | |
|  | (1) | (2) | (3) | (4) | (5) | (6) | (7) |
|  | | | | | | | |
| Social distancing | -14.581 |  |  |  |  |  |  |
|  | (13.344) |  |  |  |  |  |  |
|  |  |  |  |  |  |  |  |
| Self-isolating |  | 2.546 |  |  |  |  |  |
|  |  | (7.094) |  |  |  |  |  |
|  |  |  |  |  |  |  |  |
| Avoiding crowds |  |  | -9.219 |  |  |  |  |
|  |  |  | (13.640) |  |  |  |  |
|  |  |  |  |  |  |  |  |
| Avoiding small groups |  |  |  | -5.875 |  |  |  |
|  |  |  |  | (8.026) |  |  |  |
|  |  |  |  |  |  |  |  |
| Less in person interaction |  |  |  |  | 9.872 |  |  |
|  |  |  |  |  | (8.389) |  |  |
|  |  |  |  |  |  |  |  |
| Less overall interaction |  |  |  |  |  | -0.025 |  |
|  |  |  |  |  |  | (6.478) |  |
|  |  |  |  |  |  |  |  |
| Mask outdoors |  |  |  |  |  |  | 0.472 |
|  |  |  |  |  |  |  | (6.823) |
|  |  |  |  |  |  |  |  |
|  | | | | | | | |
| Observations | 516 | 516 | 516 | 516 | 516 | 516 | 516 |
| R^2^ | 0.003 | 0.000 | 0.001 | 0.002 | 0.004 | 0.000 | 0.000 |
| Adjusted R^2^ | -0.505 | -0.510 | -0.508 | -0.508 | -0.504 | -0.510 | -0.510 |
| F Statistic (df = 1; 341) | 1.194 | 0.129 | 0.457 | 0.536 | 1.385 | 0.000 | 0.005 |
|  | | | | | | | |

Notes: Fixed effects regression using each prescribed social behaviour (independent variable) to predict social tendency (dependent variable) in separate models. Standard errors are clustered on an individual level. * p<0.05, ** p<0.01, *** p<0.001

Robustness checks for the association between less overall interaction and sad tendency

A.9. Simple panel linear models 1-7 on sad tendency, each model focusing on a single social behaviour in isolation: fixed effects

|  | | | | | | | |
| --- | --- | --- | --- | --- | --- | --- | --- |
|  | *Dependent variable:* | | | | | | |
|  |  | | | | | | |
|  | Sad tendency | | | | | | |
|  | (1) | (2) | (3) | (4) | (5) | (6) | (7) |
|  | | | | | | | |
| Social distancing | 5.126 |  |  |  |  |  |  |
|  | (11.906) |  |  |  |  |  |  |
|  |  |  |  |  |  |  |  |
| Self-isolating |  | -9.759 |  |  |  |  |  |
|  |  | (6.184) |  |  |  |  |  |
|  |  |  |  |  |  |  |  |
| Avoiding crowds |  |  | 7.574 |  |  |  |  |
|  |  |  | (12.116) |  |  |  |  |
|  |  |  |  |  |  |  |  |
| Avoiding small groups |  |  |  | -5.575 |  |  |  |
|  |  |  |  | (7.143) |  |  |  |
|  |  |  |  |  |  |  |  |
| Less in person interaction |  |  |  |  | -10.833 |  |  |
|  |  |  |  |  | (7.467) |  |  |
|  |  |  |  |  |  |  |  |
| Less overall interaction |  |  |  |  |  | -17.517^***^ |  |
|  |  |  |  |  |  | (5.643) |  |
|  |  |  |  |  |  |  |  |
| Mask outdoors |  |  |  |  |  |  | 1.796 |
|  |  |  |  |  |  |  | (6.056) |
|  |  |  |  |  |  |  |  |
|  | | | | | | | |
| Observations | 522 | 522 | 522 | 522 | 522 | 522 | 522 |
| R^2^ | 0.001 | 0.007 | 0.001 | 0.002 | 0.006 | 0.027 | 0.000 |
| Adjusted R^2^ | -0.509 | -0.499 | -0.508 | -0.507 | -0.501 | -0.469 | -0.510 |
| F Statistic (df = 1; 345) | 0.185 | 2.490 | 0.391 | 0.609 | 2.105 | 9.637^***^ | 0.088 |
|  | | | | | | | |

Notes: Fixed effects regression using each prescribed social behaviour (independent variable) to predict social tendency (dependent variable) in separate models. Standard errors are clustered on an individual level. * p<0.05, ** p<0.01, *** p<0.001

A.10. Simple panel linear model on sad tendency: random effects

|  | | **Estimate** | **Std. Error** | | **p** | |  |
| --- | --- | --- | --- | --- | --- | --- | --- |
| (Intercept) | | 134.596 | 87.473 | | 0.124 | |  |
| Social distancing | 1.490 | | | 9.819 | | 0.879 | |
| Self-isolating | -2.281 | | | 3.881 | | 0.557 | |
| Avoiding crowds | -3.465 | | | 9.918 | | 0.727 | |
| Avoiding small groups | -0.269 | | | 5.649 | | 0.962 | |
| Less in person interaction | -4.429 | | | 6.141 | | 0.471 | |
| Less overall interaction | -10.244 | | | 4.934 | | 0.038* | |
| Mask outdoors | -2.795 | | | 3.544 | | 0.430 | |
| N | | 174 |  | |  | |  |
| Chisq | | 11.167 |  | |  | |  |
| R^2^ | | 0.021 |  | |  | |  |
| Cohen’s *d* | | 0.157 |  | |  | |  |

Notes: Random effects regression using prescribed social behaviours (independent variables) to predict sad tendency (dependent variable). Standard errors are clustered on an individual level. Cohen’s *d* value was caculated using the behaviour 6 coefficient to estimate effect size. * p<0.05, ** p<0.01, *** p<0.001

A.11. Covariate panel linear model on sad tendency **including** time non-varying variables: random effects

|  |  |  |  |  |
| --- | --- | --- | --- | --- |
|  | Estimate | Std. Error | z-value | p |
| (Intercept) | 1537.403 | 390.062 | 3.941 | 0.000*** |
| Less overall interaction | -17.521 | 2.643 | -6.628 | 0.000*** |
| Corona fear | 2.355 | 4.500 | 0.523 | 0.601 |
| Anxious | 6.768 | 5.437 | 1.245 | 0.213 |
| Happy | -14.872 | 6.657 | -2.234 | 0.025* |
| Bis | 0.413 | 3.102 | 0.133 | 0.894 |
| Bas Drive | 8.942 | 4.137 | 2.162 | 0.031* |
| Bas Reward | -0.466 | 1.809 | -0.258 | 0.797 |
| Hours away from home | 5.712 | 6.963 | 0.820 | 0.412 |
| Stressed | -19.912 | 5.568 | -3.576 | 0.000*** |
| Social Anxiety | -0.166 | 0.409 | -0.406 | 0.685 |
| Anxiety | -6.582 | 1.641 | -4.011 | 0.000*** |
| Mental Health | -12.879 | 19.362 | -0.665 | 0.506 |
| SWB | -11.722 | 3.636 | -3.224 | 0.001** |
| Extraversion | -1.315 | 1.459 | -0.901 | 0.368 |
| Conscientiousness | 5.926 | 7.191 | 0.824 | 0.410 |
| Stability | -13.774 | 8.610 | -1.600 | 0.110 |
| Agreeableness | 5.010 | 7.873 | 0.636 | 0.525 |
| Openness | -7.207 | 6.620 | -1.089 | 0.276 |
| Age | -33.277 | 13.479 | -2.469 | 0.014* |
| Gender | -81.893 | 35.112 | -2.332 | 0.020* |
| Ethnicity (prefer not to say) | -223.477 | 221.507 | -1.009 | 0.313 |
| White | -125.585 | 198.824 | -0.632 | 0.528 |
| Asian | -114.670 | 250.763 | -0.457 | 0.647 |
| Asian/Black | -261.064 | 294.797 | -0.886 | 0.376 |
| Income | 6.125 | 7.238 | 0.846 | 0.397 |
| Region | -2.451 | 4.495 | -0.545 | 0.586 |
| Cardiovascular disease | -166.668 | 88.626 | -1.881 | 0.060 |
| Diabetes | -41.742 | 70.208 | -0.595 | 0.552 |
| Chronic respiratory disease | -20.056 | 102.294 | -0.196 | 0.845 |
| Hypertension | 30.450 | 67.805 | 0.449 | 0.653 |
| Asthma | -13.148 | 67.888 | -0.194 | 0.846 |
| Other serious condition | -9.602 | 75.259 | -0.128 | 0.898 |
| No health condition | -8.253 | 72.309 | -0.114 | 0.909 |
| Children | 2.219 | 14.930 | 0.149 | 0.882 |
| House living | -1.033 | 11.384 | -0.091 | 0.928 |
| Key worker | -49.456 | 40.239 | -1.229 | 0.219 |
| Covid symptoms (none) | -175.356 | 199.242 | -0.880 | 0.379 |
| Covid Symptoms (s.o I know living apart) | -251.760 | 205.949 | -1.222 | 0.222 |
| Covid Symptoms (myself) | -88.939 | 226.682 | -0.392 | 0.695 |
| Covid Symptoms (myself + s.o I live with) | -338.779 | 225.068 | -1.505 | 0.132 |
| Covid Symptoms (s.o I know) | -203.559 | 280.723 | -0.725 | 0.468 |
| Covid Symptoms (s.o I live with) | -173.106 | 247.914 | -0.698 | 0.485 |
| N | 174 |  |  |  |
| Chisq | 112.736 |  |  |  |
| R^2^ | 0.070 |  |  |  |
| Cohen’s *d* for less overall interaction | -0.503 |  |  |  |

Notes: Random effects panel linear regression using less overall interaction and all other time varying *and* non time varying covariates (independent variables) to predict sad tendency (dependent variable). Cohen’s *d* value was caculated using the less overall interaction coefficient to estimate effect size. * p<0.05, ** p<0.01, *** p<0.001

Testing for associations between affective variables and reduced-overall-interaction

A.12. Testing for associations between trait level affective variables with reduced-overall-interaction, fixed effects panel linear model

|  | | | |
| --- | --- | --- | --- |
|  | Dependent variable: | | |
|  |  | | |
|  | Reduced-overall-interaction | | |
|  | (1) | (2) | (3) |
|  | | | |
| Anxiety | 0.009 |  |  |
|  | (0.035) |  |  |
|  |  |  |  |
| Social Anxiety |  | 0.003 |  |
|  |  | (0.009) |  |
|  |  |  |  |
| Life Satisfaction |  |  | -0.028 |
|  |  |  | (0.184) |
|  |  |  |  |
|  | | | |
| Observations | 522 | 522 | 522 |
| R^2^ | 0.0002 | 0.0004 | 0.0001 |
| Adjusted R^2^ | -0.510 | -0.510 | -0.510 |
| F Statistic (df = 1; 345) | 0.068 | 0.132 | 0.024 |
|  | | | |
| Note: | ^*^p<0.1; ^**^p<0.05; ^***^p<0.01 | | |

A.13. Testing for associations between coronavirus specific affective variables with reduced-overall-interaction, fixed effects panel linear models

|  | | |
| --- | --- | --- |
|  | Dependent variable: | |
|  |  | |
|  | Reduced-overall-interaction | |
|  | (1) | (2) |
|  | | |
| Corona fear (self) | 0.155 |  |
|  | (0.121) |  |
|  |  |  |
| Corona fear (others) |  | 0.115 |
|  |  | (0.113) |
|  |  |  |
|  | | |
| Observations | 522 | 522 |
| R^2^ | 0.005 | 0.003 |
| Adjusted R^2^ | -0.503 | -0.506 |
| F Statistic (df = 1; 345) | 1.632 | 1.031 |
|  | | |
| Note: | ^*^p<0.1; ^**^p<0.05; ^***^p<0.01 | |

A.14. Testing for associations between state level affective variables with reduced-overall-interaction, fixed effects panel linear model

|  | | | | | |
| --- | --- | --- | --- | --- | --- |
|  | Dependent variable: | | | | |
|  |  | | | | |
|  | Reduced-overall-interaction | | | | |
|  | (1) | (2) | (3) | (4) | (5) |
|  | | | | | |
| Stressed | -0.045 |  |  |  |  |
|  | (0.091) |  |  |  |  |
|  |  |  |  |  |  |
| Anxious |  | 0.142 |  |  |  |
|  |  | (0.089) |  |  |  |
|  |  |  |  |  |  |
| Happy |  |  | -0.076 |  |  |
|  |  |  | (0.129) |  |  |
|  |  |  |  |  |  |
| Valence |  |  |  | -0.009 |  |
|  |  |  |  | (0.010) |  |
|  |  |  |  |  |  |
| Arousal |  |  |  |  | -0.001 |
|  |  |  |  |  | (0.008) |
|  |  |  |  |  |  |
|  | | | | | |
| Observations | 522 | 522 | 522 | 522 | 522 |
| R^2^ | 0.001 | 0.007 | 0.001 | 0.002 | 0.00001 |
| Adjusted R^2^ | -0.509 | -0.499 | -0.509 | -0.507 | -0.510 |
| F Statistic (df = 1; 345) | 0.240 | 2.536 | 0.348 | 0.787 | 0.005 |
|  | | | | | |
| Note: | ^*^p<0.1; ^**^p<0.05; ^***^p<0.01 | | | | |

Testing for associations between affective variables and sad tendency

A.15. Testing for associations between trait level affective variables with sad tendency, fixed effects panel linear model

|  | | | |
| --- | --- | --- | --- |
|  | Dependent variable: | | |
|  |  | | |
|  | Sad tendency | | |
|  | (1) | (2) | (3) |
|  | | | |
| Anxiety | -8.703^**^ |  |  |
|  | (3.667) |  |  |
|  |  |  |  |
| Social Anxiety |  | -0.621 |  |
|  |  | (0.993) |  |
|  |  |  |  |
| Life Satisfaction |  |  | 12.193 |
|  |  |  | (19.545) |
|  |  |  |  |
|  | | | |
| Observations | 522 | 522 | 522 |
| R^2^ | 0.016 | 0.001 | 0.001 |
| Adjusted R^2^ | -0.486 | -0.508 | -0.508 |
| F Statistic (df = 1; 345) | 5.633^**^ | 0.391 | 0.389 |
|  | | | |
| Note: | ^*^p<0.1; ^**^p<0.05; ^***^p<0.01 | | |

A.16. Testing for associations between coronavirus specific affective variables with sad tendency, fixed effects panel linear model

|  | | |
| --- | --- | --- |
|  | Dependent variable: | |
|  |  | |
|  | Sad tendency | |
|  | (1) | (2) |
|  | | |
| Corona fear (self) | 3.322 |  |
|  | (12.893) |  |
|  |  |  |
| Corona fear (other) |  | -3.914 |
|  |  | (12.020) |
|  |  |  |
|  | | |
| Observations | 522 | 522 |
| R^2^ | 0.0002 | 0.0003 |
| Adjusted R^2^ | -0.510 | -0.510 |
| F Statistic (df = 1; 345) | 0.066 | 0.106 |
|  | | |
| Note: | ^*^p<0.1; ^**^p<0.05; ^***^p<0.01 | |
|  |  | |

A.17. Testing for associations between state level affective variables with sad tendency, fixed effects panel linear model

|  | Dependent variable: | | | | |
| --- | --- | --- | --- | --- | --- |
|  |  | | | | |
|  | Sad tendency | | | | |
|  | (1) | (2) | (3) | (4) | (5) |
|  | | | | | |
| Stressed | -19.030^**^ |  |  |  |  |
|  | (9.635) |  |  |  |  |
|  |  |  |  |  |  |
| Anxious |  | -9.758 |  |  |  |
|  |  | (9.494) |  |  |  |
|  |  |  |  |  |  |
| Happy |  |  | -6.046 |  |  |
|  |  |  | (13.666) |  |  |
|  |  |  |  |  |  |
| Valence |  |  |  | 0.745 |  |
|  |  |  |  | (1.060) |  |
|  |  |  |  |  |  |
| Arousal |  |  |  |  | -1.009 |
|  |  |  |  |  | (0.859) |
|  |  |  |  |  |  |
|  | | | | | |
| Observations | 522 | 522 | 522 | 522 | 522 |
| R^2^ | 0.011 | 0.003 | 0.001 | 0.001 | 0.004 |
| Adjusted R^2^ | -0.493 | -0.506 | -0.509 | -0.508 | -0.504 |
| F Statistic (df = 1; 345) | 3.901^**^ | 1.056 | 0.196 | 0.494 | 1.379 |
|  | | | | | |
| Note: | ^*^p<0.1; ^**^p<0.05; ^***^p<0.01 | | | | |

Full breakdown of participant characteristics

A.18. Proportion of participants in each age-group

| **Age group** | **Count** |
| --- | --- |
| 18-24 | 2% |
| 25-34 | 15% |
| 35-44 | 14% |
| 45-54 | 19% |
| 55-64 | 20% |
| 65+ | 30% |

A.19. Proportion of participants in each physical health group

(1 = Excellent, 5 – Poor)

| **Physical Health** | **Count** |
| --- | --- |
| 1 | 10% |
| 2 | 26% |
| 3 | 39% |
| 4 | 18% |
| 5 | 7% |

A.20. Proportion of participants in each income group

| **Income brackets** | | **Wave 1** |
| --- | --- | --- |
| Under £5000 | 6% | |
| £5000-£9999 | 9% | |
| £10,000-£14,999 | 10% | |
| £15,000-£19,999 | 9% | |
| £20,000-£24,000 | 13% | |
| 25,000-£34,999 | 23% | |
| £35,000-£44,999 | 11% | |
| £45,000-£54,999 | 9% | |
| £55,000-£99,999 | 9% | |
| £100,000+ | 3% | |
|  |  | |

Income split followed a normal distribution across income brackets with less people at the lower and higher bounds, as expected (this information was only collected in wave 1)

A.21. Proportion of participants in each UK region

|  | | **Count** |
| --- | --- | --- |
| Greater London | 15% | |
| South East | 16% | |
| South West | 8% | |
| West Midlands | 10% | |
| North West | 8% | |
| North East | 6% | |
| Yorkshire and the Humber | 5% | |
| East Midlands | 8% | |
| East Anglia | 9% | |
| Scotland | 9% | |
| Northern Ireland | 1% | |
| Wales | 4% | |

Comparisons between final sample participants and dropouts

A.22. A comparison of gender in final sample with dropouts

Full sample

| **Gender** | **Count** |
| --- | --- |
| Male | 53% |
| Female | 47% |

Dropouts

| **Gender** | **Count** |
| --- | --- |
| Male | 47% |
| Female | 53% |

A.23. A comparison of age in final sample with dropouts

Full sample

| **Age group** | **Count** |
| --- | --- |
| 18-24 | 2% |
| 25-34 | 15% |
| 35-44 | 14% |
| 45-54 | 19% |
| 55-64 | 20% |
| 65+ | 30% |

Dropouts

| **Age group** | **Count** |
| --- | --- |
| 18-24 | 3% |
| 25-34 | 14% |
| 35-44 | 17% |
| 45-54 | 21% |
| 55-64 | 17% |
| 65+ | 27% |

A.24. A comparison of income in final sample with dropouts

Full sample

| **Income brackets** | | **Wave 1** |
| --- | --- | --- |
| Under £5000 | 6% | |
| £5000-£9999 | 9% | |
| £10,000-£14,999 | 10% | |
| £15,000-£19,999 | 9% | |
| £20,000-£24,000 | 13% | |
| 25,000-£34,999 | 23% | |
| £35,000-£44,999 | 11% | |
| £45,000-£54,999 | 9% | |
| £55,000-£99,999 | 9% | |
| £100,000+ | 3% | |
|  |  | |

Dropouts

| **Income brackets** | | **Wave 1** |
| --- | --- | --- |
| Under £5000 | 8% | |
| £5000-£9999 | 8% | |
| £10,000-£14,999 | 13% | |
| £15,000-£19,999 | 10% | |
| £20,000-£24,000 | 10% | |
| 25,000-£34,999 | 13% | |
| £35,000-£44,999 | 9% | |
| £45,000-£54,999 | 6% | |
| £55,000-£99,999 | 9% | |
| £100,000+ | 3% | |
| NA | 10% | |
|  |  | |

List of figures

A.1. SCRT instructions


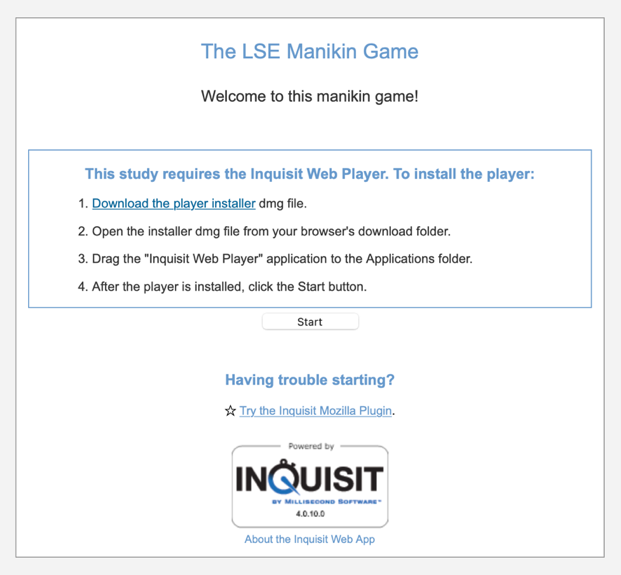


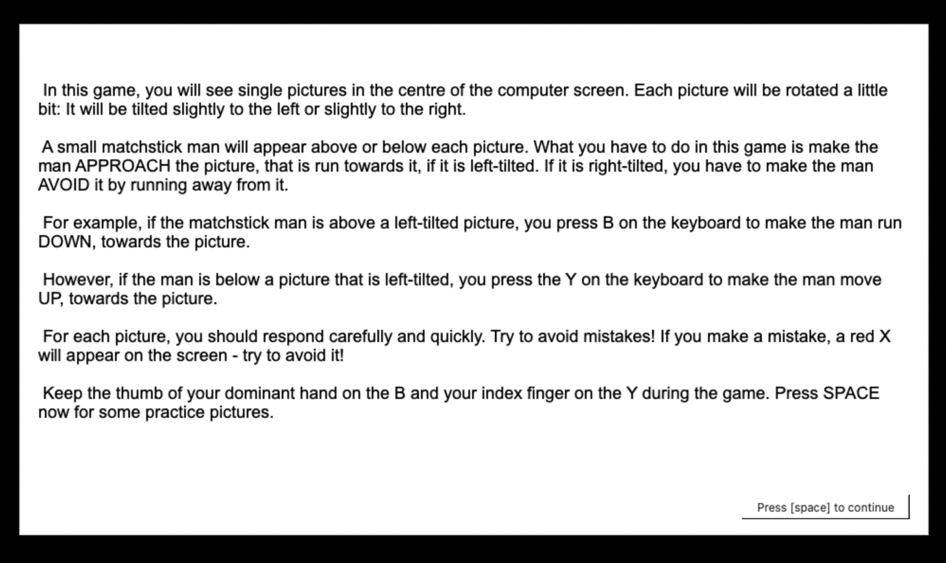


Sample size calculation

Considering that we could not identify appropriate statistical packages that could calculate power for the exact panel linear models we aimed to use, we computed several power analyses for Person correlations, given that they are broadly comparable to our panel models. In line with current practices in the field of psychology, we first calculated the sample size needed to detect a medium effect size (r = .30), assuming the alpha level of 0.05 and the power level of 0.80 (Faul et al., 2009). The analysis indicated that 82 participants would need to be tested. We then ran the same analysis but changed the alpha level to a more conservative 0.001. In this case, the sample size requirement was 179. Overall, this analysis indicated that testing roughly 179 participants would be sufficient to detect medium effects, regardless of whether a standard (0.05) or more conservative (0.001) alpha level is used. Given that we eventually obtained a comparable sample size (i.e., 174), we then computed sensitivity power analyses (Faul et al., 2009) to identify the smallest effect size that could be reliable obtained with the sample size we recruited, assuming the power of 0.80. These analyses showed that the study is likely to be sufficiently powered to detect effect size *r* = 0.21 (assuming the alpha level of 0.05) and effect size *r* = 0.30 (assuming the alpha level of 0.001). Therefore, it is plausible that the present research was well powered to detect at least medium effect sizes, regardless of the alpha level used.

Faul, F., Erdfelder, E., Buchner, A., & Lang, A. G. (2009). Statistical power analyses using G* Power 3.1: Tests for correlation and regression analyses. *Behavior research methods*, *41*(4), 1149-1160.

Data access

Data files for this this study and associated coding guidelines can be found here <https://osf.io/ydqgm/>
